# Assisted reproductive technology in Japan: A summary report for 2021 by the Ethics Committee of the Japan Society of Obstetrics and Gynecology

**DOI:** 10.1002/rmb2.12552

**Published:** 2023-12-30

**Authors:** Yukiko Katagiri, Seung Chik Jwa, Akira Kuwahara, Takeshi Iwasa, Masanori On, Keiichi Kato, Hiroshi Kishi, Yoshimitsu Kuwabara, Fuminori Taniguchi, Miyuki Harada, Akira Iwase, Yutaka Osuga

**Affiliations:** ^1^ Department of Obstetrics and Gynecology, Faculty of Medicine Toho University Tokyo Japan; ^2^ Department of Obstetrics and Gynecology Jichi Medical University Tochigi Japan; ^3^ Department of Obstetrics and Gynecology, Graduate School of Biomedical Sciences Tokushima University Tokushima Japan; ^4^ Department of Obstetrics and Gynecology Tokyo Medical University Tokyo Japan; ^5^ Kato Ladies Clinic Tokyo Japan; ^6^ Department of Obstetrics and Gynecology The Jikei University School of Medicine Tokyo Japan; ^7^ Department of Obstetrics and Gynecology Nippon Medical School Tokyo Japan; ^8^ Department of Obstetrics and Gynecology Tottori University Faculty of Medicine Tottori Japan; ^9^ Department of Obstetrics and Gynecology, Graduate School of Medicine The University of Tokyo Tokyo Japan; ^10^ Department of Obstetrics and Gynecology Gunma University Graduate School of Medicine Maebashi Japan

**Keywords:** assisted reproductive technologies, fertility rate, in vitro fertilization, intracytoplasmic sperm injections, Japan

## Abstract

**Purpose:**

The Japan Society of Obstetrics and Gynecology (JSOG) registry gathers comprehensive data from registered assisted reproductive technology (ART) facilities in Japan. Herein, we report 2021 ART cycle characteristics and outcomes.

**Methods:**

Descriptive statistics were used to summarize and analyze 2021 data.

**Results:**

In 2021, 625 ART facilities participated in the registry; 27 facilities did not conduct ART cycles and 598 registered treatment cycles. In total, 498 140 cycles were registered, and there were 69 797 neonates (increases of 10.7% and 15.5%, respectively, from the previous year). The number of freeze‐all in vitro fertilization (IVF) and intracytoplasmic sperm injection (ICSI) cycles decreased in 2021; the number of neonates born was 2268 for IVF‐embryo transfer (ET) cycles and 2850 for ICSI cycles. Frozen–thawed ET (FET) cycles increased markedly from 2020 (11.2% increase). In 2021, 239 428 FET cycles were conducted, resulting in 87 174 pregnancies and 64 679 neonates. For fresh transfers, the total single ET, singleton pregnancy rate, and singleton live birth rates were 82.7%, 97.0%, and 97.3%; for FET, these rates were 84.9%, 96.9%, and 97.1%.

**Conclusions:**

The 2021 Japanese ART registry analysis showed marked increases in both total treatment cycles and live births from the previous year.

## Graphical abstract

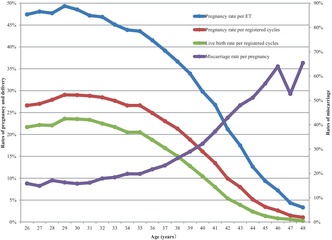

## 1 INTRODUCTION

The well‐known issues of rapid aging in Japan, the increasing trend in later childbearing,^1^ the declining marriage trend,^2^, ^3^ and their impact on fertility rates continue to be considerable challenges.^4^, ^5^ According to the Ministry of Health, Labour and Welfare, the fertility rate in Japan decreased to 1.33 in 2020, which is lower than the previous record low of 1.36 reported in 2019. This is a significant drop from the 1.44 fertility rate reported in 2016.^6^ The World Bank reported a global fertility rate of 2.4 in 2019, which decreased by 0.10 in 2020, at 2.3, indicating not only a marked difference from Japan's current rate,^7^ but also depicting a similar global trend in declining fertility rates.^7^

To address the effects of aging and the potential shortage of human capital caused by Japan's aging population and decreased fertility rates, policy interventions have been created to boost fertility rates.^4^ The most relevant interventions have been increases in the national childcare capacity and gradual expansion of parental leave rights,^4^ increases in the financial accessibility to fertility treatments for patients,^8^ and expansion of government subsidies for assisted reproductive technology (ART) since January 2021 before the insurance coverage for ART came into effect in April 2022,^9^ among others.

Japan remains among the top countries globally in providing women with fertility treatments.^10^, ^11^ In 2020,^12^ the number of treatment cycles (449 900 treatment cycles) and 60 381 live births resulting from ART reflected a 1.79% and 0.36% decrease, respectively, from that reported in 2019.^13^ The Japan Society of Obstetrics and Gynecology (JSOG) has been using the ART registry system since 1986 to collect data on national trends of ART use and outcomes, as well as the online registration system implemented in 2007, both of which were established with the intent of aiding the understanding of the current effectiveness of ART, ensuring its safety, and making informed decisions related to ART in Japan.^12^ This report aims to provide updated data on the characteristics and outcomes of registered ART cycles during 2021 and to compare the present results with results from previous years.

## 2 MATERIALS AND METHODS

### 2.1 Data source and data collection

The JSOG registry collects comprehensive data from registered ART facilities across Japan on demographic and background characteristics of patients, clinical information including infertility diagnosis, complications caused by treatment, obstetric history and outcomes, and ART cycle‐specific data.^14^ Registered facilities adhered to the same standard definitions of parameters and outcomes and submitted their data to JSOG annually using standard formats and channels. In case of missing data, JSOG made the necessary inquiries to obtain the data. The present retrospective descriptive analysis investigated registered cycle characteristics and treatment outcomes using data from the Japanese ART registry in 2021 with a cutoff date of November 30, 2022. The JSOG Ethics Committee approved this analysis and reporting of these data.

### 2.2 Variables of interest

Data were collected for the number of registered cycles, oocyte retrievals, embryo transfer (ET) cycles, freeze‐all‐embryo/oocyte cycle, pregnancies, and number of neonates by fertilization method (in vitro fertilization [IVF], intracytoplasmic sperm injection [ICSI], and frozen–thawed embryo transfer [FET]) and were analyzed and compared with data from previous years. Characteristics of registered cycles and pregnancy outcomes were described for fresh and FET cycles. Fresh cycle data were stratified by fertilization method (i.e., IVF, ICSI, gamete intrafallopian transfer [GIFT], oocyte freezing, and others).

### 2.3 Outcomes

The treatment outcomes analyzed and compared were defined as follows. Pregnancy was defined as the confirmation of a gestational sac in utero. Miscarriage was the spontaneous or unplanned loss of a fetus from the uterus before 22 weeks of gestation. Live birth was defined as the delivery of at least one live neonate after 22 weeks of gestation. Multiple pregnancy rates were also calculated.

The pregnancy outcomes analyzed and compared included ectopic pregnancy, heterotopic pregnancy, artificially induced abortion, stillbirth, and fetal reduction. Pregnancy, live birth, miscarriage, and multiple pregnancy rates were also analyzed by patient age. Treatment outcomes for FET cycles using frozen–thawed oocytes were also analyzed.

### 2.4 Statistical analysis

All analyses were conducted using the STATA MP statistical package, version 17.0 (Stata, College Station). As this study focuses on descriptive analysis, statistical testing was not conducted.

## 3 RESULTS

In 2021, 625 ART facilities, of 625 registered with JSOG, participated in this year's registry, of which 27 facilities did not implement ART activities, and the remaining 598 facilities registered treatment cycles.

Table 1 summarizes the main trends in the numbers of registered cycles, egg retrievals, pregnancy, and neonate births categorized by IVF, ICSI, and FET cycles in Japan between 2007 and 2021. In 2021, 498 140 cycles were registered, and 69 797 neonates were recorded (10.7% and 15.5% increases, respectively, compared with the previous year). Notably, the number of registered IVF and ICSI cycles increased by 8.7% and 12.3%, respectively, from the previous year. The number of freeze‐all IVF decreased by 1.2% and ICSI by 0.80% in 2021, and the number of neonates born was 2268 for IVF‐ET cycles and 2850 for ICSI cycles, the first decreased slightly from the previous year (0.6%) and the latter increased by 9.8%. The number of FET cycles has increased continuously since 2007, and this trend was maintained in 2021; however, the increase from 2020 was 11.2%, a large increase compared with the 0.04% increase in 2019 and 5.8% from 2018 to 2019. In 2021, the number of FET cycles was 239 428, resulting in 87 174 pregnancies and 64 679 neonates (14.4% and 16.5% increases, respectively).

**TABLE 1:** Trends in numbers of registered cycles, oocyte retrieval, pregnancy, and neonates based on IVF, ICSI, and frozen–thawed embryo transfer cycles in Japan, 2007–2021.

Year	IVF^a^						ICSI^b^						FET cycle^c^			
	No. of registered cycles	No. of egg retrievals	No. of freeze‐all cycles	No. of ET cycles	No. of cycles with pregnancy	No. of neonates	No. of registered cycles	No. of egg retrievals	No. of freeze‐all cycles	No. of ET cycles	No. of cycles with pregnancy	No. of neonates	No. of registered cycles	No. of ET cycles	No. of cycles with pregnancy	No. of neonates
2007	53 873	52 165	7626	28 228	7416	5144	61 813	60 294	11 541	34 032	7784	5194	45 478	43 589	13 965	9257
2008	59 148	57 217	10 139	29 124	6897	4664	71 350	69 864	15 390	34 425	7017	4615	60 115	57 846	18 597	12 425
2009	63 083	60 754	11 800	28 559	6891	5046	76 790	75 340	19 046	35 167	7330	5180	73 927	71 367	23 216	16 454
2010	67 714	64 966	13 843	27 905	6556	4657	90 677	88 822	24 379	37 172	7699	5277	83 770	81 300	27 382	19 011
2011	71 422	68 651	16 202	27 284	6341	4546	102 473	100 518	30 773	38 098	7601	5415	95 764	92 782	31 721	22 465
2012	82 108	79 434	20 627	29 693	6703	4740	125 229	122 962	41 943	40 829	7947	5498	119 089	116 176	39 106	27 715
2013	89 950	87 104	25 085	30 164	6817	4776	134 871	134 871	49 316	41 150	8027	5630	141 335	138 249	45 392	32 148
2014	92 269	89 397	27 624	30 414	6970	5025	144 247	141 888	55 851	41 437	8122	5702	157 229	153 977	51 458	36 595
2015	93 614	91 079	30 498	28 858	6478	4629	155 797	153 639	63 660	41 396	8169	5761	174 740	171 495	56 888	40 611
2016	94 566	92 185	34 188	26 182	5903	4266	161 262	159 214	70 387	38 315	7324	5166	191 962	188 338	62 749	44 678
2017	91 516	89 447	36 441	22 423	5182	3731	157 709	155 758	74 200	33 297	6757	4826	198 985	195 559	67 255	48 060
2018	92 552	90 376	38 882	20 894	4755	3402	158 859	157 026	79 496	29 569	5886	4194	203 482	200 050	69 395	49 383
2019	88 074	86 334	40 561	17 345	4002	2974	154 824	153 014	83 129	24 490	4789	3433	215 203	211 758	74 911	54 188
2020	82 883	81 286	42 530	13 362	3094	2282	151 732	150 082	87 697	19 061	3626	2596	215 285	211 914	76 196	55 503
2021	88 362	86 901	42 016	13 219	3115	2268	170 350	168 659	86 992	19 740	3875	2850	239 428	236 211	87 174	64 679

Abbreviations: ET, embryo transfer; FET, frozen–thawed embryo transfer; GIFT, gamete intrafallopian transfer; ICSI, intracytoplasmic sperm injection; IVF, in vitro fertilization.

^a^ Including GIFT and other.

^b^ Including split‐ICSI cycles.

^c^ Including cycles using frozen–thawed oocyte.

**FIGURE 1:**
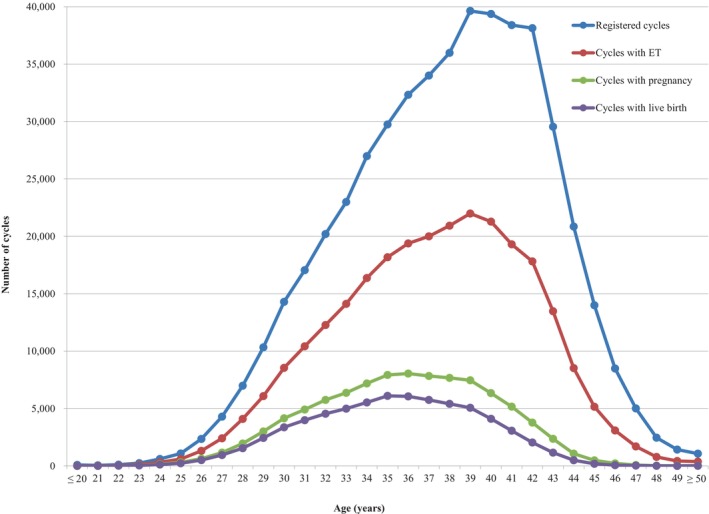
Distribution of maternal age from all registered cycles, cycles for ET, cycles leading to pregnancy, and cycles leading to live births in 2021. Adapted from the Japan Society of Obstetrics and Gynecology ART Databook 2021 (https://www.jsog.or.jp/activity/art/2021_JSOG‐ART.pdf). ET, embryo transfer.

Figure 1 shows the age distributions for all registered cycles and different subgroups of cycles for ET, pregnancy, and live births in 2021. The mean patient age for registered cycles was 37.8 years (standard deviation [SD] ± 4.8); the mean age for pregnancy and live birth cycles was 35.8 years (SD ± 4.2) and 35.3 years (SD ± 4.1), respectively. In 2021, 39.9% of ART cycles registered were undertaken for women aged 40 years or over.

### 3.1 Treatment and pregnancy outcomes

The detailed characteristics and treatment outcomes of registered fresh cycles are shown in Table 2. In 2021, 81 116 IVF cycles, 31 661 split‐ICSI cycles, 136 661 ICSI cycles using ejaculated spermatozoa, 2028 ICSI cycles using testicular sperm extraction (TESE), 10 GIFT cycles, 1103 cycles for oocyte freezing, and 6133 other cycles were registered. In total, 255 560 cycles resulted in oocyte retrieval, of which 129 008 (50.4%) were freeze‐all cycles. The pregnancy rate per ET cycle of IVF was 23.6%, and for ICSI using ejaculated spermatozoa was 18.8%. The total single ET rate was 82.7%, and the pregnancy rate following a single ET cycle was 21.7%. Live birth rates per ET were 16.7% for IVF, 18.6% for split‐ICSI, 13.3% for ICSI using ejaculated spermatozoa, 6.3% for ICSI with TESE, and 20.0% for GIFT. There were 6634 singleton pregnancies and 4845 singleton live births. The rate of singleton pregnancies was 97.0%, and the rate of singleton live births was 97.3%. In total, 1103 cycles for oocyte freezing were registered, and 1084 oocyte retrievals were conducted. Of these, 830 cycles led to successfully frozen oocytes.

**TABLE 2:** Characteristics and treatment outcomes of registered fresh cycles in assisted reproductive technology in Japan, 2021.

Variables	IVF	Split‐ICSI	ICSI		GIFT	Frozen oocyte	Other^a^	Total
			Ejaculated sperm	TESE				
No. of registered cycles	81 116	31 661	136 661	2028	10	1103	6133	258 712
No. of egg retrievals (zero or more)	79 795	31 441	135 194	2024	10	1084	6012	255 560
No. of fresh ET cycles (one or more)	12 763	3203	16 217	320	10	0	446	32 959
No. of freeze‐all cycles	38 323	21 866	64 003	1123	0	830	2863	129 008
No. of cycles with pregnancy	3011	790	3040	45	4	0	100	6990
Pregnancy rate per ET	23.6%	24.7%	18.8%	14.1%	40.0%		22.4%	21.2%
Pregnancy rate per egg retrieval	3.8%	2.5%	2.3%	2.2%	40.0%		1.7%	2.7%
Pregnancy rate per egg retrieval excluding freeze‐all cycles	7.3%	8.3%	4.3%	5.0%	40.0%		3.2%	5.5%
SET cycles	11 067	2764	12 804	199	1		417	27 252
Pregnancy following SET cycles	2630	707	2436	34	0		96	5903
Rate of SET cycles	86.7%	86.3%	79.0%	62.2%	10.0%		93.5%	82.7%
Pregnancy rate following SET cycles	23.8%	25.6%	19.0%	17.1%	0.0%		23.0%	21.7%
Miscarriages	742	154	760	21	2		20	1699
Miscarriage rate per pregnancy	24.6%	19.5%	25.0%	46.7%	50.0%		20.2%	24.3%
Singleton pregnancies^b^	2870	756	2869	39	2		98	6634
Multiple pregnancies^b^	82	20	97	1	2		1	203
Twin pregnancies	80	19	94	1	2		1	197
Triplet pregnancies	2	1	3	0	0		0	6
Quadruplet pregnancies	0	0	0	0	0		0	0
Multiple pregnancy rate	2.8%	2.6%	3.3%	2.5%	50.0%		1.0%	3.0%
Live births	2133	595	2152	20	2		78	4980
Live birth rate per ET	16.7%	18.6%	13.3%	6.3%	20.0%		17.5%	15.1%
Total no. of neonates	2188	610	2220	20	2		78	5118
Singleton live births	2078	581	2086	20	2		78	4845
Twin live births	55	13	64	0	0		0	132
Triplet live births	0	1	2	0	0		0	3
Quadruplet live births	0	0	0	0	0		0	0
Ectopic pregnancies	38	12	52	2	0		0	104
Heterotopic pregnancies	0	0	0	0	0		0	0
Artificial abortions	19	6	14	1	0		0	40
Still births	14	1	8	0	0		0	23
Fetal reductions	1	0	2	0	0		0	3
Cycles with unknown pregnancy outcomes	45	19	35	1	0		1	101

Abbreviations: ET, embryo transfer; GIFT, gamete intrafallopian transfer; ICSI, intracytoplasmic sperm injection; IVF, in vitro fertilization; SET, single embryo transfer; TESE, testicular sperm extraction; ZIFT, zygote intrafallopian transfer.

^a^ Others include ZIFT.

^b^ Singleton, twin, triplet, and quadruplet pregnancies were defined on the basis of the number of gestational sacs in utero.

Table 3 summarizes the characteristics and treatment outcomes of FET cycles. In 2021, a total of 239 048 cycles were registered. Of these, 238 049 were registered as FET cycles. Of the latter, 235 156 FETs were actually conducted. With a pregnancy rate of 36.9%, FET cycles resulted in 86 841 pregnancies. FET cycles resulted in 21 548 miscarriages. The miscarriage rate per pregnancy was 24.8%, the same as in the previous year, and the live birth rate per FET increased to 26.6% from 25.5% observed in 2020. The single ET rate was 84.9%, slightly lower than in 2020 (85.1%), resulting in a slightly increased pregnancy rate of 38.1% from 37.1%. The singleton pregnancy rate was 96.9%, and the live birth rate was 97.1%.

**TABLE 3:** Characteristics and treatment outcomes of frozen cycles in assisted reproductive technology in Japan, 2021.

Variables	FET	Other^a^	Total
No. of registered cycles	238 049	999	239 048
No. of FET	235 156	849	236 005
No. of cycles of pregnancy	86 841	290	87 131
Pregnancy rate per FET	36.9%	34.2%	36.9%
SET cycles	199 698	701	200 399
Pregnancy following SET cycles	76 054	252	76 306
Rate of SET cycles	84.9%	82.6%	84.9%
Pregnancy rate following SET cycles	38.1%	36.0%	38.1%
Miscarriages	21 548	75	21 623
Miscarriage rate per pregnancy	24.8%	25.9%	24.8%
Singleton pregnancies^b^	82 932	272	83 204
Multiple pregnancies^b^	2619	8	2627
Twin pregnancies	2567	7	2574
Triplet pregnancies	48	1	49
Quadruplet pregnancies	4	0	4
Multiple pregnancy rate	3.1%	2.9%	3.1%
Live births	62 619	206	62 825
Live birth rate per FET	26.6%	24.3%	26.6%
Total no. of neonates	64 436	212	64 648
Singleton live births	60 818	200	61 018
Twin live births	1785	6	1791
Triplet live births	16	0	16
Quadruplet live births	0	0	0
Ectopic pregnancies	467	1	468
Heterotopic pregnancies	22	0	22
Artificial abortions	405	2	407
Stillbirths	237	0	237
Fetal reductions	25	0	25
Cycles with unknown pregnancy outcomes	1327	3	1330

Abbreviations: FET, frozen–thawed embryo transfer; SET, single embryo transfer.

^a^ Including cycles using frozen–thawed oocytes.

^b^ Singleton, twin, triplet, and quadruplet pregnancies were defined on the basis of the number of gestational sacs in utero.

### 3.2 Outcomes by patient age

**FIGURE 2:**
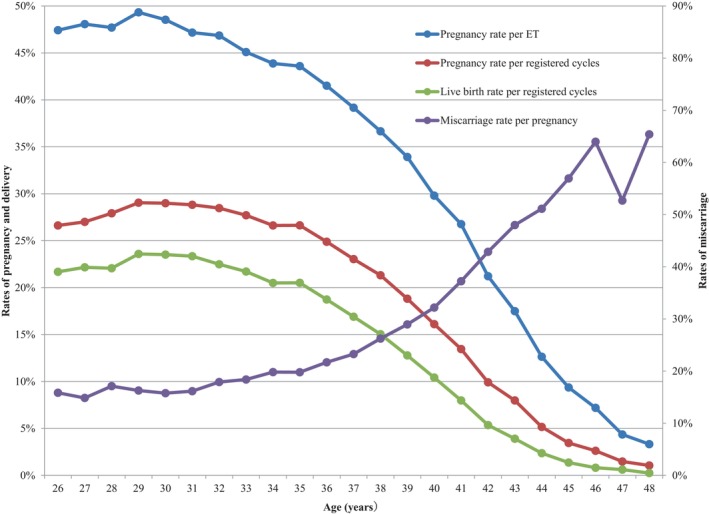
Pregnancy, live birth, and miscarriage rates according to patient age in all registered cycles in 2021. Adapted from the Japan Society of Obstetrics and Gynecology ART Databook 2021 (https://www.jsog.or.jp/activity/art/2021_JSOG‐ART.pdf). ET, embryo transfer.

Figure 2 shows the pregnancy, live birth, and miscarriage rates by patient age in all registered cycles in 2021. Of note, the pregnancy rate per ET was above 45% until approximately 32 years of age, with a progressive decline from that point onward, becoming even more marked beyond the age of 40 years. Similar trends were observed for pregnancy and live birth rates. Conversely, miscarriage rates remained below 20% before 35 years of age, and increased subsequently and progressively until the late 40s.

Table 4 shows the treatment outcomes of registered cycles by patient age in Japan in 2021. The pregnancy rate per ET exceeded 40% for maternal ages between 21 and 36 years. Gradual decreases in pregnancy rates per ET were observed with increasing maternal age, starting at age 26 years. Rates fell below 30% for women aged >40 years, below 20% among women aged >43 years of age, below 10% for women aged >45 years, and <5% for women aged >47 years. The miscarriage rates were below 20% for all women between 22 and 35 years of age and increased gradually with increasing maternal age. Women in their early 40s had miscarriage rates of between 32.2% and 51.1%, while women in their mid‐40s had miscarriage rates over 52%. Live birth rates per registered cycle were between 15.0% and 23.6% for women between 22 and 38 years of age, which declined sharply to below 15.0% at 39 years of age and below 10.0% among women >41 years of age.

**TABLE 4:** Treatment outcomes of registered cycles based on patient age in Japan, 2021.

Age (years)	No. of registered cycles	No. of ET cycles	No. of cycles with pregnancy	Multiple pregnancies	Miscarriage	Cycles with live birth	Pregnancy rate/registered ET (%)	Pregnancy rate/registered cycles (%)	Live birth rate/registered cycles (%)	Miscarriage rate /pregnancy (%)	Multiple pregnancy rate (%)^a^
≤20	90	12	3	0	1	2	25.00	3.30	2.20	33.30	0.00
21	54	18	9	0	4	4	50.00	16.70	7.40	44.40	0.00
22	105	48	23	0	3	18	47.90	21.90	17.10	13.00	0.00
23	240	128	59	1	8	49	46.10	24.60	20.40	13.60	1.75
24	611	322	158	5	28	123	49.10	25.90	20.10	17.70	3.18
25	1083	598	312	16	63	231	52.20	28.80	21.30	20.20	5.26
26	2348	1318	625	27	99	509	47.40	26.60	21.70	15.80	4.38
27	4291	2411	1159	26	172	951	48.10	27.00	22.20	14.80	2.29
28	6986	4090	1951	57	334	1541	47.70	27.90	22.10	17.10	2.97
29	10 333	6084	3001	79	488	2437	49.30	29.00	23.60	16.30	2.66
30	14 294	8539	4145	119	653	3361	48.50	29.00	23.50	15.80	2.91
31	17 057	10 424	4917	159	794	3983	47.20	28.80	23.40	16.10	3.28
32	20 202	12 273	5751	182	1030	4543	46.90	28.50	22.50	17.90	3.21
33	22 986	14 121	6368	189	1171	4990	45.10	27.70	21.70	18.40	3.01
34	26 983	16 372	7184	191	1422	5530	43.90	26.60	20.50	19.80	2.70
35	29 751	18 178	7925	250	1568	6104	43.60	26.60	20.50	19.8%	3.20
36	32 326	19 383	8046	274	1745	6055	41.50	24.90	18.70	21.70	3.45
37	34 011	20 004	7836	259	1822	5747	39.20	23.00	16.90	23.30	3.36
38	35 988	20 924	7670	223	2011	5412	36.70	21.30	15.00	26.20	2.95
39	39 631	21 988	7457	219	2158	5066	33.90	18.80	12.80	28.90	2.99
40	39 376	21 281	6341	202	2040	4106	29.80	16.10	10.40	32.20	3.24
41	38 403	19 302	5167	153	1924	3067	26.80	13.50	8.00	37.20	3.01
42	38 130	17 816	3780	86	1620	2045	21.20	9.90	5.40	42.90	2.32
43	29 547	13 482	2359	71	1132	1155	17.50	8.00	3.90	48.00	3.07
44	20 850	8517	1076	28	550	492	12.60	5.20	2.40	51.10	2.65
45	13 999	5160	483	13	275	192	9.40	3.50	1.40	56.90	2.74
46	8490	3086	222	4	142	69	7.20	2.60	0.80	64.00	1.86
47	5012	1697	74	0	39	31	4.40	1.50	0.60	52.70	0.00
48	2466	782	26	1	17	6	3.30	1.10	0.20	65.40	4.00
49	1428	432	20	0	16	4	4.60	1.40	0.30	80.00	0.00
≥50	1069	380	17	0	7	10	4.50	1.60	0.90	41.20	0.00
Total	498 140	269 170	94 164	2834	23 336	67 833	35.00	18.90	13.60	24.80	3.06

Abbreviation: ET, embryo transfer.

^a^ Multiple pregnancies were defined on the basis of the number of gestational sacs in utero.

### 3.3 Treatment outcomes for FET cycles using frozen–thawed oocytes

In 2021, 380 cycles using frozen–thawed oocytes were registered in Japan, of which 206 FETs were actually implemented. Forty‐three pregnancies were achieved, with a pregnancy rate per FET of 20.9% and a live birth rate of 13.6%. The miscarriage rate per pregnancy was 32.6% (Table 5).

**TABLE 5:** Treatment outcomes of embryo transfers using frozen–thawed oocyte in assisted reproductive technology in Japan, 2021.

Variables	Embryo transfers using frozen–thawed oocytes
No. of registered cycles	380
No. of ET	206
No. of cycles with pregnancy	43
Pregnancy rate per ET	20.9%
SET cycles	133
Pregnancy following SET cycles	26
Rate of SET cycles	64.6%
Pregnancy rate following SET cycles	19.6%
Miscarriages	14
Miscarriage rate per pregnancy	32.6%
Singleton pregnancies^a^	38
Multiple pregnancies^a^	4
Twin pregnancies	4
Triplet pregnancies	0
Quadruplet pregnancies	0
Multiple pregnancy rate	9.5%
Live births	28
Live birth rate per ET	13.6%
Total number of neonates	31
Singleton live births	25
Twin live births	3
Triplet live births	0
Quadruplet live births	0
Ectopic pregnancies	1
Intrauterine pregnancies coexisting with ectopic pregnancy	0
Artificial abortions	0
Still births	0
Fetal reductions	0
Cycles with unknown pregnancy outcomes	0

Abbreviations: ET, embryo transfer; SET, single embryo transfer.

^a^ Singleton, twin, triplet, and quadruplet pregnancies were defined on the basis of the number of gestational sacs in utero.

## 4 DISCUSSION

This report provides an overview of the characteristics and outcomes of ART cycles registered in the Japanese ART registry system during 2021. We also compare the present results with those from 2020^12^ and previous years.^13^, ^15^, ^16^, ^17^ The data were collected from 598 ART facilities registered with JSOG in Japan.

In 2021, a total of 498 140 cycles were registered with JSOG in Japan, resulting in 69 797 neonates. These figures represent an increase in both measures by 10.7% and 15.5%, respectively, compared with the previous year's data. The number of fresh cycles, including IVF and ICSI, also showed an increase from the data recorded in the previous year, with increases of 8.7% and 12.3%, respectively, contrasting with previously observed trends. However, the number of freeze‐all IVF cycles decreased by 1.2%, while ICSI cycles decreased by 0.8%. The number of neonates born from IVF‐ET cycles slightly reduced by 0.6%, while for ICSI cycles, it increased by 9.8%. FET cycles increased by 11.2% in 2021, resulting in 239 428 cycles, 87 174 pregnancies, and 64 679 neonates.

Conducting this annual analysis is crucial to comprehend the changing trends and patterns in ART in Japan. This information is essential, given the continuously declining fertility rate, the growing elderly population—especially in Japan—and the decreasing population growth worldwide.^2^, ^18^, ^19^, ^20^ ART data reporting is considered a vital part of the health care infrastructure as it helps us assess the extent to which data are collected. Furthermore, these data may serve as quality‐of‐care indicators and inform evidence‐based policies that promote positive health outcomes through clinical practices in infertility treatment.^21^

In Japan, subsidies were previously available for ART, instead of being covered by health insurance. These subsidies varied across different municipalities; some municipalities provided additional subsidies for patients.^22^ However, there were income restrictions, and only patients with an annual household income <7 300 000 JPY could receive the subsidies. In April 2020, it was decided that insurance would cover ART, and subsidies would be abolished. This new measure was scheduled to take effect by April 2022. Between January 2021 and April 2022, while ART insurance coverage had not yet been implemented, the government expanded the fertility treatment subsidy system. They removed income restrictions and increased the subsidized amount to reduce the financial burden of infertility treatment.^23^ As ART insurance coverage has been recently implemented, its effects will likely be more apparent in subsequent reports. However, the present results for 2021 show an increase in the overall number of cases of infertility treatment. This may be a result of the expanded subsidies and removal of income limitations, as well as a greater participation of younger populations.

Other factors could have potentially affected ART use during the study period despite the measures taken. The elevated ART costs and income disparities^8^, ^24^, ^25^ are relevant factors, as it has been recently reported that women from higher income households had a higher probability of seeking assistance for fertility problems despite the financial support and increased insurance coverage. For example, some treatment options may not be covered, but mixed medical care using both insurance coverage and private expenses is prohibited. Thus, patients with higher incomes may opt to pursue all the infertility treatment out of pocket to have more treatment options, if needed.^26^ The social distancing measures applied during the coronavirus disease (COVID‐19) pandemic delayed important events such as weddings, as well as subsequent childbearing, particularly for young couples.^27^ Furthermore, because of the COVID‐19 pandemic, the government extended the age limitation for receiving subsidies (age 43 to age 44 years, and age 40 to age 41 years, respectively).^28^ The Japanese Society for Reproductive Medicine also recommended postponing all infertility treatments, including ART in April of 2020 and issued another statement recommending the resumption of treatments with measures to control infection. Altogether, these measures resulted in the shift to receiving ART treatment from 2020 to 2021, with particularly low treatment cycles in April and May of 2020.^29^ It has also been reported that individuals were cautious regarding vaccination safety and its potential effects on pregnancy and infants. Currently, no evidence supports the idea that SARS‐CoV‐2 vaccination negatively affects semen parameters or spermatogenesis, ovarian function, ovarian reserve, or folliculogenesis. Most studies have not reported a significant impact of COVID‐19 vaccination on ART outcomes.^30^, ^31^, ^32^ It has also been reported that administering intravenous immunoglobulin G (IVIG) for immune‐related infertility treatment does not affect the efficacy of COVID‐19 vaccines. Therefore, COVID‐19 vaccines can be given during ART cycles that involve the use of IVIG.^33^

Although the rate of freeze‐all cycles continuously increased up to 2020, that rate first decreased in both IVF and ICSI cycles in 2021. This may reflect that patients tended to not avoid pregnancy compared in 2020; in 2020, because of the COVID‐19 pandemic, many patients and clinicians chose freeze‐all cycles and avoided pregnancy because of the lack of evidence regarding the safety of pregnancy during the pandemic, resulting in a relatively high freeze‐all rate (51.3% in IVF cycles and 57.8% in ICSI cycles).

There is a heavy mental health and physical burden associated with ART.^10^, ^34^ Cultural and social factors include the social stigma associated with infertility.^35^, ^36^, ^37^ Unmarried couples in Japan usually do not have children.^38^ Further measures that could help improve Japan's current ART and fertility trends can be summarized under the governmental movements to support infertility patients. This act facilitates continuous consultation support, infertility support networks, and peer support groups, especially for those who have experienced miscarriages.

The study's strengths and limitations have been previously reported.^12^ The most relevant strengths were that the data from registered ART facilities nationwide must provide annual reports, resulting in high reporting compliance. Standardizing procedures and definitions for cycle‐specific information across registered ART facilities also reduced reporting bias. Regarding limitations, some data for which collection is not standardized, such as background information, and information on newer treatments, may be incomplete or missing. To address this, information on such variables is being updated in registries from January 2022 onward.

Based on the 2021 data analysis conducted by the Japanese ART registry under the JSOG, there has been a marked increase in the number of ART cycles and neonates born compared with 2020. The trend of increasing numbers of FETs continued throughout 2021, with more significant increases compared with the previous year. The total single ET rate for fresh transfers was 82.7%, with singleton pregnancy and live birth rates of 97.0% and 97.3%, respectively. For FET, the single ET rate was 84.9%, leading to a singleton pregnancy rate of 96.9% and live birth rates of 97.1%. In summary, the 2021 data analysis from the Japanese ART registry administered by the JSOG showed promising results, with a high success rate for ART cycles resulting in live births.

## CONFLICT OF INTEREST STATEMENT

“Iwase, Akira” is an Editorial Board member of Reproductive Medicine and Biology and a coauthor of this article. To minimize bias, they were excluded from all editorial decision‐making related to the acceptance of this article for publication. The authors have no conflict of interest to disclose in relation to this work.

## HUMAN RIGHTS STATEMENTS AND INFORMED CONSENT

All procedures were performed in accordance with the ethical standards of the relevant committees on human experimentation (institutional and national) and the Helsinki Declaration of 1964 and its later amendments.

## ANIMAL RIGHTS

This report does not contain any studies performed by any authors that included animals.
